# Detection of Foreign Matter in Transfusion Solution Based on Gaussian Background Modeling and an Optimized BP Neural Network

**DOI:** 10.3390/s141119945

**Published:** 2014-10-24

**Authors:** Fuqiang Zhou, Zhen Su, Xinghua Chai, Lipeng Chen

**Affiliations:** School of Instrumentation Science and Optoelectronics Engineering, Beihang University, XueYuan Road No. 37, Haidian District, Beijing 100191, China; E-Mails: 13401166133@126.com (Z.S.); cxh88_88@163.com (X.C.); clp386@163.com (L.C.)

**Keywords:** detection of foreign matter, Gaussian background model, mind evolutionary algorithm, BP neural network, ReliefF feature selection

## Abstract

This paper proposes a new method to detect and identify foreign matter mixed in a plastic bottle filled with transfusion solution. A spin-stop mechanism and mixed illumination style are applied to obtain high contrast images between moving foreign matter and a static transfusion background. The Gaussian mixture model is used to model the complex background of the transfusion image and to extract moving objects. A set of features of moving objects are extracted and selected by the ReliefF algorithm, and optimal feature vectors are fed into the back propagation (BP) neural network to distinguish between foreign matter and bubbles. The mind evolutionary algorithm (MEA) is applied to optimize the connection weights and thresholds of the BP neural network to obtain a higher classification accuracy and faster convergence rate. Experimental results show that the proposed method can effectively detect visible foreign matter in 250-mL transfusion bottles. The misdetection rate and false alarm rate are low, and the detection accuracy and detection speed are satisfactory.

## 1. Introduction

Medical transfusion is one of the important preparations of the pharmaceutical industry in China. At present, the transfusion packaging market mainly consists of plastic bottles, glass bottles and soft bags. The sales of plastic bottles have increased quickly, because of their advantages of good chemistry stability, light weight and lesser chance of being contaminated. However, some visible foreign matter may mix in the transfusion solution during the process of producing, filling and packaging. This foreign matter can cause serious diseases [1], such as tumors, phlebitis, anaphylactic reaction or even death, when they are injected into the blood of patients. Thus, the transfusion product must undergo a rigorous inspection before entering the market. However, due to a lack of pivotal technologies, most of the pharmaceutical manufacturers in China have adopted the light inspection method. In a dark room, the trained inspectors reverse a transfusion container illuminated by a bright lamp to check whether visible foreign matter exists and to decide if the product is qualified or not subjectively. The method is simple, but the judgment lacks a rigorous and objective evaluation criteria. Therefore, it has low accuracy and repeatability.

A visual inspection system for foreign matter in injection products was developed early [2]. Additionally, a lot research has been done, and many methods [3–5] have been proposed. Most of the research is aimed at ampoules or vials. They are rotated at a high speed and are stopped abruptly. The solution in the container forms a vortex due to inertia. The foreign matter moving with the solution can be distinguished from the stationary background on the surface of the ampoule. However, an ampoule or a vial is small, and the shape of is standard circular. Additionally, it has a smooth, highly transparent surface. Therefore, it is easy to gather the foreign matter in the center of the container and to distinguish it from the simple background. In this paper, the detected object is a 250-mL plastic container filled with sodium chloride injection. The transparency of a plastic bottle is lower compared to a glass one, and the surface of it is easily formed into a large area for the diffuse reflection region. As the cross-sections of the bottle are elliptical, they have a complicated surface, such as embossed symbols and graduations on the surface and paper labels, which illustrates the usage and dosage, product batch number and other information. All of these characteristics described above increase the difficulty of detecting and identifying foreign matter in this complex circumstance.

The detection of foreign matter mixed in a plastic bottle of medicinal solution using real-time video image processing is proposed in [6], but this method cannot distinguish foreign matter and bubbles effectively; it just tries to obtain high contrast images between foreign matter and bubbles by adjusting the positions, angles and the exposure time of the light source. A method has been proposed in [7], which use mean shift to track only falling moving objects in five frames as foreign matter. This method may have a high misdetection rate for some lighter density foreign matter, such as floating debris, which is circling with the solution, occasionally rising, occasionally falling, and may be missed. Moghadas *et al.* [8] proposed a non-rotating method, which was based on stereo vision, to acquire moving objects and used a Multi-layer Perceptron (MLP) neural network to distinguish foreign matter and bubbles in a vial. However, this method may be confused by spurious light and the instability of the light source. A method based on the frame difference method and the modified pulse-coupled neural networks segmentation method [9] is used in the process of moving object detection. The difference method is commonly used, but this method weakens the energy of foreign matter and is sensitive to noise and spurious light, which may extract interference points in a poor quality image. Therefore, it has high requirements for subsequent segmentation algorithms.

In this paper, Gaussian background modeling is used to extract moving objects. It is a good method for complex background modeling. It models each pixel according to the gray value and can separate the moving objects and static background more effectively, even for poor quality image, compared with the difference method. After that, a set of features is extracted and selected for each moving object by the ReliefF algorithm. Then BP neural network is used as a classifier to train the features to distinguish foreign matter and bubbles. The BP neural network is one of the most widely used neural network models. It can learn and store a large amount of input-output models mapping relationships. However, the BP algorithm has the shortcomings of slow convergence, low accuracy and easily to falls into the local minimum solution. Therefore, the mind evolutionary algorithm (MEA) is applied to optimize the BP neural network. MEA is a global optimization approach. It improves search efficiency and solves the earliness and slow convergence speed in BP successfully. The experimental results show that our method can detect and identify visible foreign matter effectively with satisfactory speed and accuracy.

This article is organized as follows. In Section 2, we describe the system framework and illumination style. In Section 3, we demonstrate the key algorithm of detection and identification. The Gaussian background modeling method is applied to detect foreign matter firstly, and it can accurately extract moving objects while suppressing background noise. In the detection process, bubbles are a significant external interference for foreign matter. Thus, we apply the BP neural network to train corresponding features to distinguish between foreign matter and bubbles. To reduce the computation of the system, optimal feature vectors are obtained by the ReliefF features selection algorithm. The BP algorithm optimized by MEA can have a higher classification accuracy and faster convergence rate. In Section 4, we present the experimental results and analysis. Finally, conclusions are drawn in Section 5.

## 2. System Framework and Illumination Style

### 2.1. System Framework

When the transfusion bottle is still, the foreign matter is often down in the bottom or adhered to the side walls of the bottle. In order to obtain high contrast images, firstly the transfusion bottle starts to rotate, driven by the motor at 7 r/s speed, and then, it decelerates to 3 r/s to produce a stationary vortex and is stopped suddenly.

**Figure 1.**
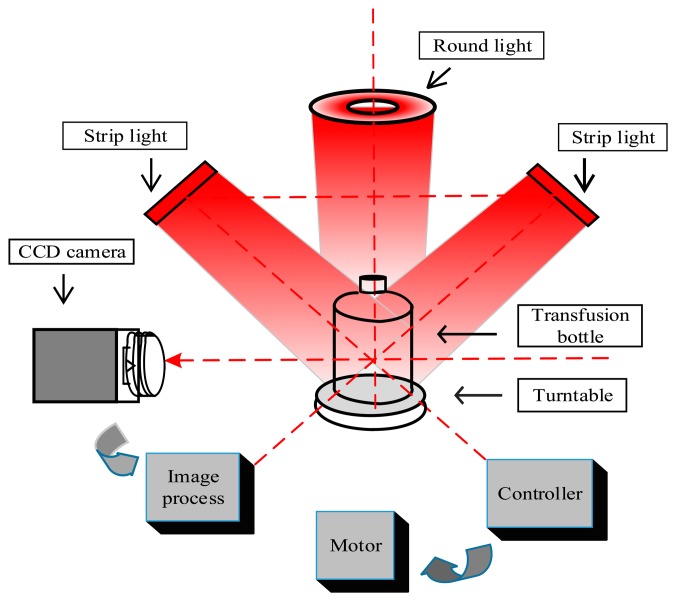
The framework of the detection system for a plastic transfusion bottle.

To reduce the interference of bubbles, the camera and light source are activated simultaneously after the bubbles rise to the liquid surface (about 0.7 s) to acquire a sequence of foreign matter images. At last, the images are processed by the image process unit to judge whether the product contains foreign matter or not. The framework of the detection system for a plastic transfusion bottle is shown in Figure 1.

### 2.2. Illumination Style

To enhance the contrast between the moving foreign matter and the static background, a proper mixed illumination style is designed. Two bar-shaped LED lights are mounted at the left and right sides of the transfusion bottle, diagonally above 45 degrees, the center line of the two cluster beams intersects at the center of the transfusion bottle.

This method produces a uniform stable light background and avoids diffuse reflectance when light is shining directly on the side walls of the bottle. Another round condensing LED light is installed above the bottle to enhance the reflection of foreign matter. The whole transfusion bottle is illuminated uniformly; the foreign matter image is black on the CCD camera because of its obstruction of the transmitted light. The refraction light is reflected by the white matter images on the CCD. The mixed illumination style can obtain a high contrast images of foreign matter. In the experiment, the red LED light is used because of its sensitivity to tiny foreign matter in the solution.

## 3. Key Algorithm of Detection and Identification

A transfusion bottle has a complicated surface, such as scales, scratches and embossed labels on the surface, which makes it difficult to distinguish foreign matter and an uneven surface. In the experiment, the bottle's complicated surface, system random noise, the instability of light source and the dithering of mechanical devices form a complex background. To achieve the detection of foreign matter, a Gaussian mixture model is applied to model a transfusion image. It models each pixel according to the gray value and updates the model parameters to separate a moving object and the static background. In the detection process, bubbles are a significant external interference for foreign matter. Thus, after the detection, a set of features is extracted for each moving object and selected by the ReliefF algorithm. The ReliefF algorithm is applied to assign corresponding weights for each feature according to its contribution to the classification performance. At last, the BP neural network is used as a classifier to train the extracted features to achieve classification and identification between foreign matter and bubbles. To improve the speed of convergence and to increase the accuracy of classification, MEA is applied to optimize the connection weights and thresholds of the BP neural network. In the experiment, rubber, hairs and floating debris as foreign matter are distinguished from bubbles.

**Figure 2.**
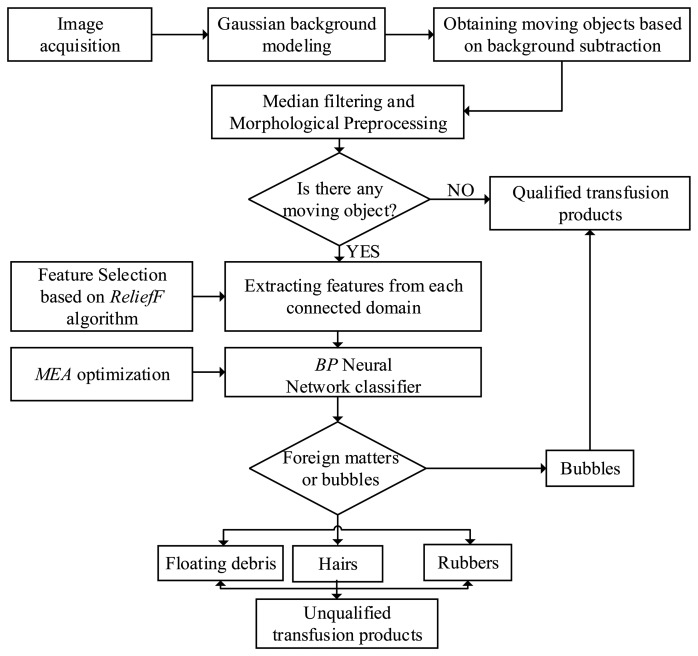
Detection and identification flowchart of foreign matter in transfusion. MEA, mind evolutionary algorithm; BP, back propagation.

The detection and identification flowchart of foreign matter in transfusion is shown in Figure 2.

### 3.1. Moving Object Detection in Transfusion

**Figure 3.**
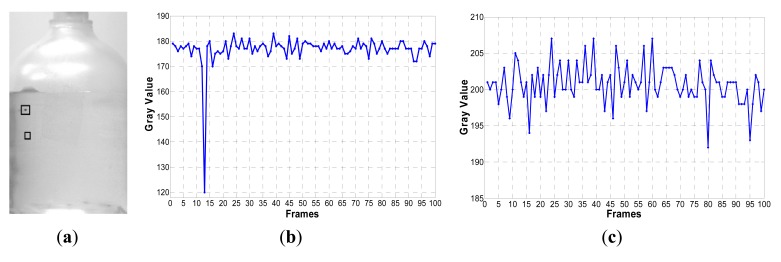
Variation curve of the gray value at the same position of sequential images. (**a**) A transfusion image with a black foreign matter; (**b**) The variation curve of the gray value at the position [136, 495] in 100 sequential images; (**c**) The variation curve of the gray value at the position [171, 636] in 100 sequential images.

In a transfusion image with foreign matter, the gray value changes of the background are relatively small without the occurrence of foreign matter. When black foreign matter or white matter appears at a pixel in the image, the gray value plunges or surges, as shown in Figure 3.

When black foreign matter appears at the position [136, 495] in Figure 3a, in the thirteenth frame image of a sequence of 100 images, the gray value plunges to 50, as shown in Figure 3b, while at the position [171, 636] in Figure 3a, without the occurrence of foreign matter, the variation is less than 15, because of the disturbance by the light source, as shown in Figure 3c. Gaussian background modeling [10–12] distinguishes a moving target from the background based on the gray variation at the same position.

(1) Based on the above analysis, we use K = 3 Gaussian models to model every pixel in the transfusion image. At time *t*, the probability of observing a background pixel *X*_t_ is the weighted sum of three Gaussian distributions:
  (1) $$p(X_{t})=\sum_{i=1}^{k}w_{i,t}*\eta (X_{t},u_{i,t},\sum_{i.t})$$
  The probability density function of the *i*-th Gaussian model is:
  (2) $$\eta_{i}(X_{t},u_{i,t},\sum_{i,t})=\frac{1}{{\left(2\pi \right)}^{\overset{n}{\;}/\underset{2}{\;}}{\left|\sum \right|}^{\frac{1}{2}}}e^{\_\frac{1}{2}{\left(X_{t}-u_{t}\right)}^{T}\sum^{-1}\left(X_{t}-u_{t}\right)},1\leq i\leq k$$
  where *w_i,t_*,*u_i,t_* ∑*_i,t_* is the weight, mean value and the corresponding covariance matrix of the *i*-th Gaussian distribution.
(2) Gaussian model parameters update: When a new image is obtained, for every pixel *X_t_*, using it to match with the already existing K Gaussian distributions, if a match is found, *X_t_* is used to update the mean and variance of the model and to increase the value of the weights of the model. If no match is found, *X_t_* is used to generate a new Gaussian distribution model to replace the already existing model with the smallest weight value.
  The criterion of a match is used as:
  (3) $$M_{i,t}=\left\{\begin{array}{cc} 1, & if\left|X_{t}-u_{i,t}\right|<\varphi \sigma_{i,t}\\ 0, & \text{otherwise} \end{array}\right\}$$
  where *φ* has an experiential value of 2.5.
(3) The judgment of foreground and background: If a pixel *X_t_* is of the background, the Gaussian distribution corresponding to *X_t_* has a smaller variance and a larger weight value. Each Gaussian model is sorted by descending order according to:
  (4) $$R_{i,t}=\overset{w_{i,t}}{\;}/\underset{\sigma_{i,t}}{\;}$$
  The background distribution is always on the top of the K distributions, and the front *n* distributions represent the background.
  (5) $$B=\arg\underset{n}{\min}\left(\sum_{k=1}^{n}w_{k}>T_{R}\right)$$
  The parameter *n* is the optimal distribution of the quantities. In this paper, the value of *T_R_* is 0.25.
  The threshold value *T_R_* represents the background distribution of weights and accounts for the smallest proportion overall. If *T_R_* is small enough, then the background model becomes single-mode, and the distribution of the background can save the amount of calculation. If *T_R_* is relatively large, then the hybrid model can accommodate the disturbance and noise of the background with a large amount of calculation. Considering the processing effects and the amount of calculation, this parameter *T_R_* in this paper is chosen as 0.25.

### 3.2. Feature Extraction of Moving Objects

For the residual images after the Gaussian background modeling, every connected domain is labeled from one to k and corresponds to a moving target. For further classification and identification of moving targets, a set of features are extracted and are fed into the BP neural network. For the tiny moving objects in a transfusion image, which has a low signal-to-noise ratio, its available features are very limited: just a few shape features, gray values and its statistical properties. The extracted features between different classes should have a great difference, and it should have little change for transformations, such as motions, rotations and translations in sequential frames for the same moving object. Based on the above analysis, a feature set is described.

Two types of region descriptors computed from the connected region have been used: the geometric invariants and the gray feature descriptors.

(1) The geometric invariants descriptors:
  The Hu [13] invariants are good descriptors for moving objects in a sequential images, since they are invariant to rotation, scaling and translation. Those invariants can be seen as nonlinear combinations of complex geometric moments:
  (6) $$u_{pq}=\sum_{x}\sum_{y}{\left(x-\overset{-}{x}\right)}^{p}{\left(y-\overset{-}{y}\right)}^{q}f(x,y)$$
  where *x* and *y* are the coordinates of the image *f*(*x*,*y*), and *x̄*, *ȳ* are the mean value of *x*, *y*. The parameters *p* and *q* are the orders of *u_pq_*.
  When the foreign matter moves with the solution, its shape may change a lot for some soft materials, such as hairs and floating debris. The Hu moments are particularly useful in the case of changes. The area *A*, the length *L* and the width of objects *W* are used as a measure of the moving object's size. The ratio of the width to length of the minimum bounding rectangle of object *R_W_*_/_*_L_* are also extracted as a reference. The ratio equals one for circular objects, such as the bubbles.
(2) The gray feature descriptors:
  When foreign matter in the solution is illuminated from above, the gray feature is a good measure for four classes of moving objects. For opaque substances, like hairs and rubber, the gray values are relatively low. The transparent bubbles have a higher gray value. Therefore, a gray feature set based on the statistical properties of the gray histogram is extracted.
  The image histogram reflects the image gray level distribution, and the expression is:
  (7) $$p(\text{k})=\frac{n_{k}}{N}(\text{k}=0,1,...,L-1)$$
  where *k* is a variable representing the gray level in an image and *N* is the total number of pixels in a gray image. Additionally, *n_k_* is the number of pixels of each gray level. The parameter *L* is the max number of gray levels.
  The feature set has fifteen features as follows (Table 1):

### 3.3. Feature Selection by the ReliefF Algorithm

The ReliefF [14–16] algorithm is a typical feature selection algorithm with high efficiency. When dealing with multi-class problems, ReliefF randomly selects an instance *R_i_* and then searches for *k* of its nearest neighbors from the same class *H_j_* and also *k* nearest neighbors from the different class *M_j_*. It updates the quality estimation weight *W*[A] for all attributes *A* depending on their values for *R_i_*, *H_j_* and *M_j_*. The entire process is repeated *m* times. Selection of *k* hits and misses ensures greater robustness of the algorithm concerning noise. The parameter *k* controls the locality of the estimates.

Ultimately, the features with greater weights serve as effective features for the classification and recognition.

### 3.4. BP Algorithm Optimized by MEA

The BP neural network is used as classifier to train the features to achieve classification and recognition between foreign matter and bubbles. The BP neural network is one of the most widely used neural network models. The BP algorithm has the shortcomings of slow convergence, low accuracy and easily falls into the local minimum solution.

**Figure 4.**
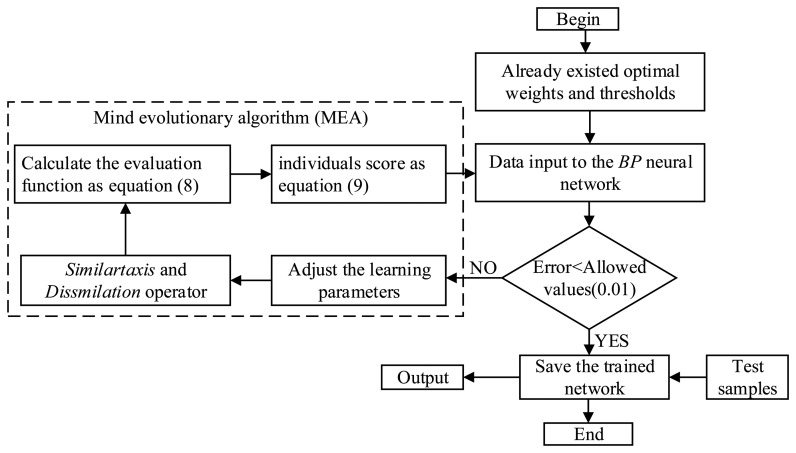
The flow chart of the BP algorithm optimized by MEA.

MEA [17–19] is a global optimization approach. It replaces crossover and mutation in the genetic algorithm (GA) with similar taxis and dissimilation, respectively. The colony is separated into some groups, and individuals exchange information, study mutually and compete locally; then, groups also exchange information, study mutually and compete globally. This algorithm improves search efficiency and solves the earliness and slow convergence speed in BP successfully. Thus, we applied the MEA to optimize the connection weights and thresholds of the BP neural network. The flow chart of the BP algorithm optimized by MEA is shown in Figure 4.

The evaluation function:

(8) $$\xi =\frac{1}{2S}\sum_{n=1}^{s}\sum_{j=1}^{q}{\left(d_{n,j}-y_{n,j}\right)}^{2}$$

where *d_n,j_* is the actual output and *y_n,j_* is the expected output.

The individuals score as:

(9) $$f(\xi )=\frac{1}{\xi }$$

## 4. Experimental Results and Analysis

**Figure 5.**
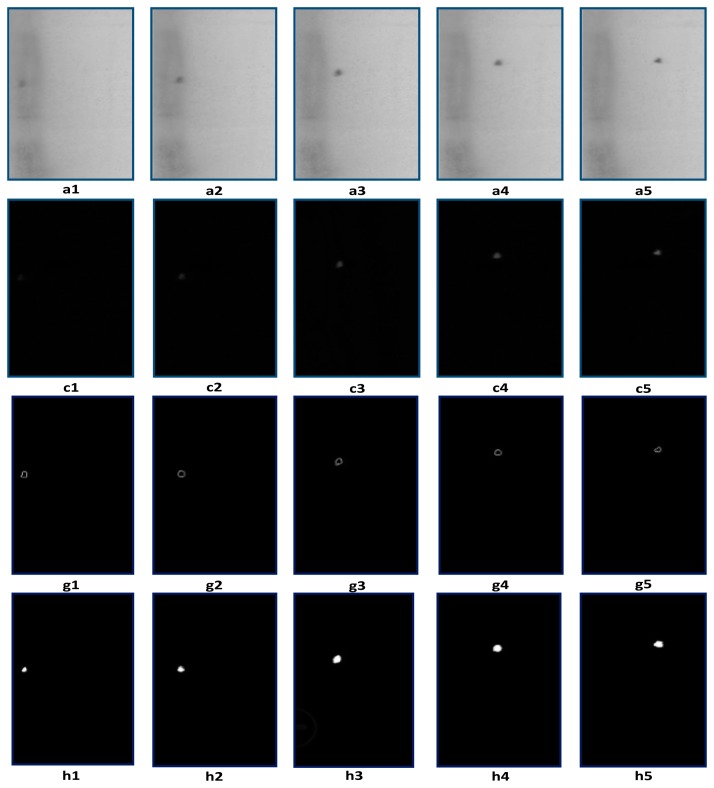
Foreign matter detection results comparison in the liquid region. (**a1–a5**) Five sequential transfusion images; (**c1–c5**) Detection results with inter-frame difference; (**g1–g5**) Edge extraction results with the Canny operator after inter-frame difference; (**h1–h5**) Detection results with the Gaussian background model.

We capture five consecutive frames of 250-mL transfusion images using a mixed illumination as mentioned above and compare the results using the Gaussian background model method and the traditional inter-frame difference method, which is shown in Figure 5. In the difference process, the energy of the moving objects is weakened, which makes it difficult to see the moving objects clearly. In order to highlight the contours of moving objects, the Canny operator is used to extract edges in the difference images, which is shown in Figure 5(g1–g5). The detection results with the Gaussian background model are shown in Figure 5(h1–h5).

Optimal Gaussian model parameters are given in experiments: the number of the Gaussian distributions *k* is tree; the initial variance is 15; the learning rate *α* is 0.05; the variance threshold *φ* is 2.5; the foreground threshold *T_R_* is 0.25.

### 4.1. Experiment 1: Foreign Matter Detection in the Liquid Region

Due to the filtering process, the edges of Figure 5(c1–c5) are blurred, and the results of Figure 5(g1–g5) and Figure 5(h1–h5) seem to be expanded, compared with the original images.

Two experiments are implemented according to the different positions where the foreign matter occurs. When foreign matter is suspended in the liquid, the foreign matter image is clear. The Canny edge detection method after inter-frame difference and the Gaussian background model method both can detect the foreign matter effectively, as shown in Figure 5.

### 4.2. Experiment 2: Foreign Matter Detection in the Reflective Regions of the Plastic Bottle

**Figure 6.**
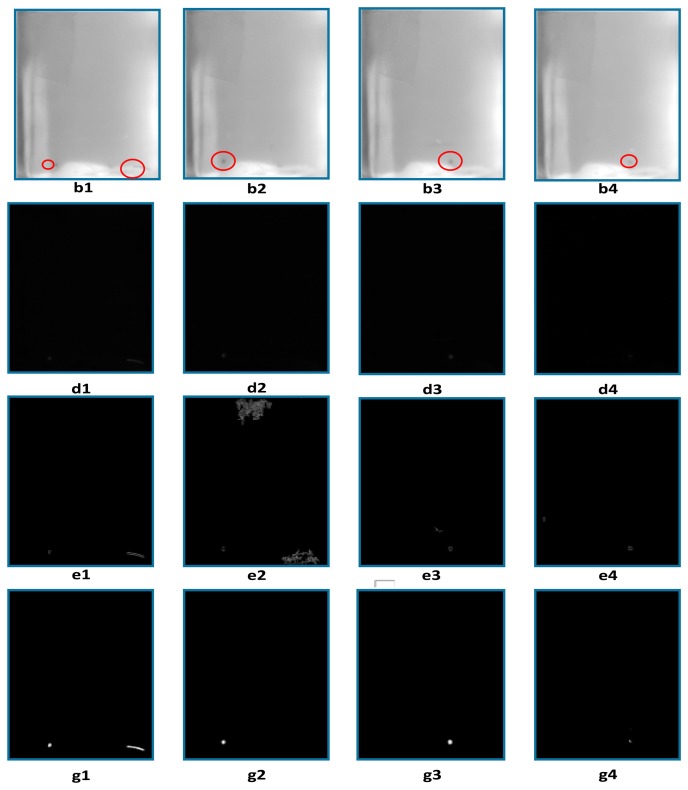
Foreign matter detection results comparison in the reflective regions of the plastic transfusion bottle. (**b1–b4**) Four frames transfusion images; (**d1–d4**) Detection results with the inter-frame difference method; (**e1–e4**) Edge extraction results with the Canny operator after the inter-frame difference method; (**g1–g4**) Detection results with the Gaussian background model method.

When foreign matter appears in the reflective regions in the bottom of the bottle, the visible foreign matter is difficult to detect after the inter-frame difference method, as shown in Figure 6(d1–d4), because its energy is weakened deeply and it is easily drowned out by the complex background. Edge detection results Figure 6(e1–e4) shows that background noise and moving objects remain in the difference images. The Gaussian background modeling method can extract the moving objects accurately and suppress background noises effectively, as shown in Figure 6(g1–g4).

The traditional frame difference method is simple and fast, but in the difference process, it would seriously weaken the energy of moving objects. For the foreign matter in the transfusion solution, it is tiny and has low energy, and the results of the difference method make it easier to be submerged in the interference and noise, especially in a bad quality image. For the use of the difference method, the subsequent processing algorithms need to enhance the energy of moving objects. A very complex segmentation method, such as modified pulse-coupled neural networks [9], need to be used to extract foreign matter, which increases the complexity of the algorithm.

The Gaussian background modeling method can update the background model in a timely manner based on the variances of the gray value in the image. It can be capable of dealing with shadows and lighting changes, slow-moving objects and objects being introduced or removed from the scene. It can perfectly extract the moving objects with its energy unchanged and does not need a subsequent complex segmentation algorithm. Traditional difference methods typically fail in these general situations. Furthermore, Gaussian background modeling is a robust, effective method for the detection of foreign matter in the transfusion solution.

### 4.3. Experiment 3: ReliefF Algorithm Features Selection

Proficient workers selected 300 bottles of transfusion products with typical foreign matter and 100 bottles of transfusion products without foreign matter, which are used to obtain the bubble samples. Each transfusion product is checked, and five images are captured in our experimental platform. After that, all of the images are processed with our algorithm, and all moving objects as samples are separated out. We pick out samples with various sizes and morphologies as the training set. We randomly select samples as the test set from the remaining samples.

**Figure 7.**
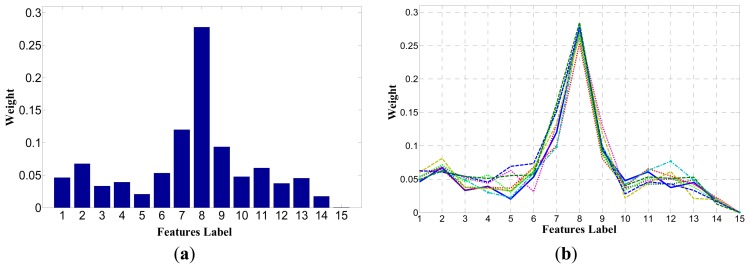
(**a**) The weight distribution of 15 features in one set; (**b**) The weight distribution of 15 features in ten sets.

In the experiment, fifteen features of ten training sets are used to test the ReliefF algorithm. Each training set has N = 200 samples, which includes four types of the objects of interest, rubber, hairs, floating debris and bubbles. Each instance searches for *k* = 8 hits and misses, and the entire algorithm is repeated m = 20 times to update the quality estimation weight. The final weight distribution result is the average result of 20 iterations. The final results showed a strong similarity, and the weights of each feature have little change, as shown in Figure 7.

In Figure 7a shows the weight distribution of fifteen features in one set. Figure 7b shows the weight distribution of fifteen features in ten sets.

The ReliefF algorithm assigns larger weights for the seventh, eighth and ninth features. Namely, the first, second and third components of Hu moments play a more important role in the classification process. While the ReliefF algorithm assigns smaller weights for others features, several are less than 0.05, which illustrates that these features are basically redundant in the classification process. Since the ReliefF algorithm cannot remove redundant features, the BP algorithm is used to test these features to remove redundant features and to obtain the optimal feature vectors.

### 4.4. Experiment 4: BP Algorithm Test for Optimal Feature Dimensionality

**Figure 8.**
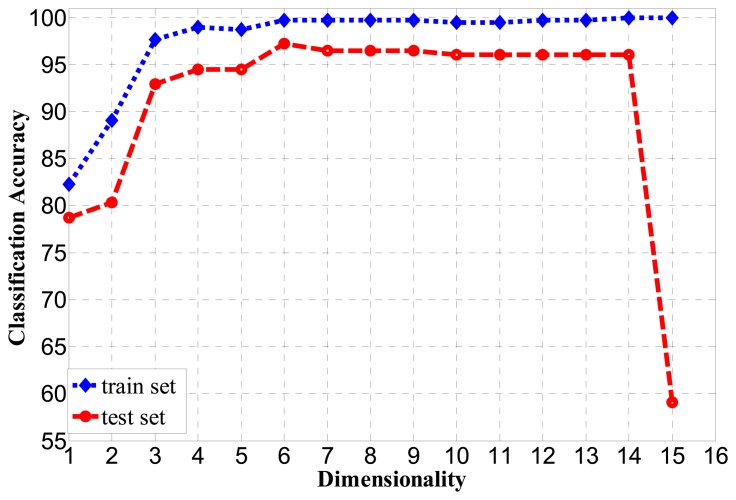
Classification accuracy according to the increased feature dimensionality.

In the test of classification accuracy, each feature is added as the weight value in descending order as shown in Figure 7. That is, the feature numbers, 7, 8, 9, 2, 11, 6, 13, 10, 1, 4, 12, 3, 5, 14 and 15, are successively added and tested. The subspace feature dimension goes from one to 15, and the BP algorithm is applied to classify the moving objects. The BP algorithm uses a three-layer neural network, and the number of hidden neurons is set to five. A training set and a test set are used, and they respectively have 300 and 200 samples. The classification accuracy is shown in Figure 8 for *m*=1,……,15 The best classification accuracy for the training set is 100% and for the test set is 97.2% for *m*=6. However, the classification accuracy is almost constant for *m*=7 ∼ 14 and greatly reduced for *m*=15. The features corresponding to *m*=7 ∼ 15 dimensions have a smaller and negative contributions for classification accuracy. In order to reduce the computation and to accelerate the speed of the system, ultimately, we removed nine redundant features. Therefore, the subspace dimension is fixed to *m*=6. In that case, the selected features are the four components of the Hu moments, *H*_1_,*H*_2_,*H*_3_,*H*_5_, the gray variance *σ*^2^ and entropy *e*.

**Figure 9.**
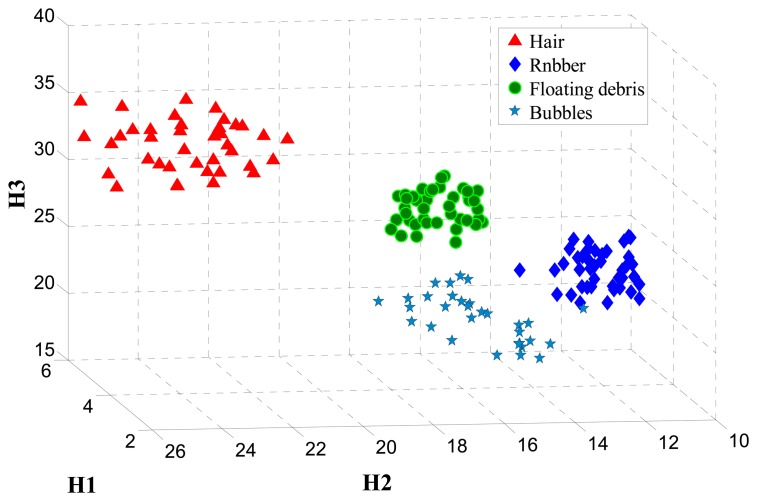
Spatial distribution of moving objects in the three-dimensional subspace.

Figure 9 represents the spatial distribution of moving objects in the best three-dimensional subspaces obtained with the feature extraction. All four classes of objects can be easily distinguished. It is clear that the distribution for rubber in the three-dimensional subspaces *H*_1_,*H*_2_,*H*_3_ is closest, because its shape is stable in the transfusion solution. While for the hairs, floating debris and bubbles, the shapes are easily changed during the process of moving with the solution, so the spatial distribution is relatively discrete, as shown in Figure 9.

### 4.5. Experiment 5: Test for BP Algorithm Optimized by MEA

In the experiment, MEA is used to optimize the connection weights and thresholds for the BP algorithm to obtain a faster convergence speed.

**Figure 10.**
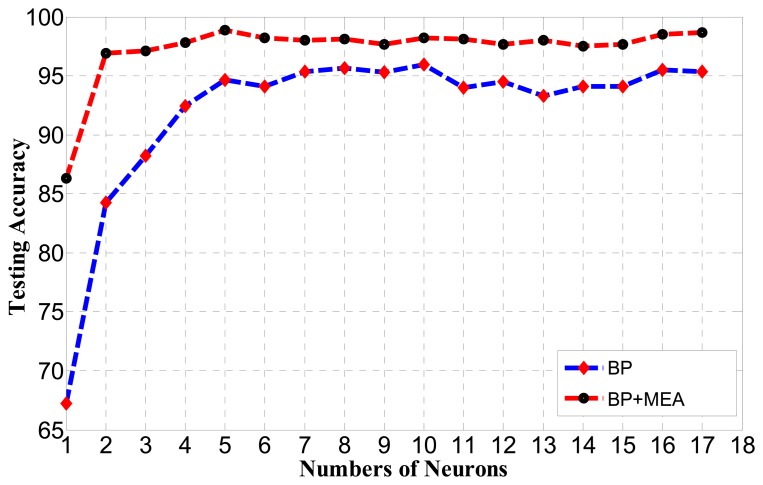
Testing accuracy comparison between BP and BP optimized by MEA for various numbers of hidden neurons.

For the BP algorithm, the choice of the number of hidden neurons is important. If the number is too small, it cannot achieve high classification and recognition accuracy. If the number is too large, the training time will increase and the network will be over-trained. To find out the best testing accuracy, the number of hidden neurons of the BP neural network is tested in Figure 10.

As observed from Figure 10, the best performance obtained by the BP algorithm is 95.68% when the number of hidden neurons is 10, while the best performance obtained by the BP algorithm optimized by MEA is 98.76% when the number is five.

**Figure 11.**
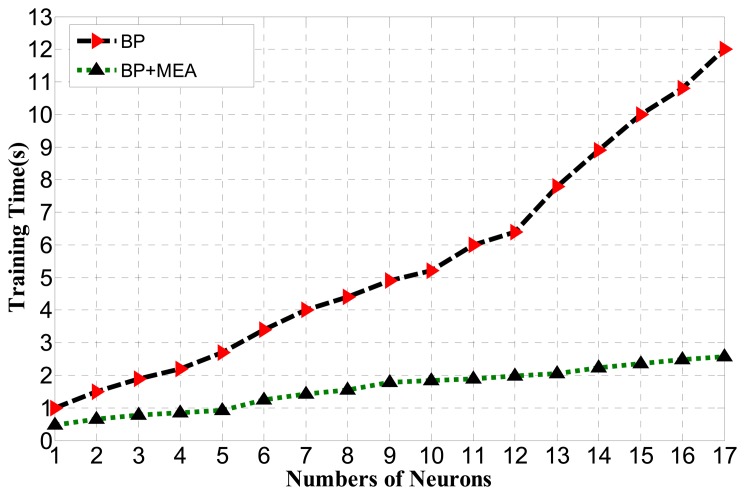
Training time comparison between BP and BP optimized by MEA for various numbers of hidden neurons.

Thus, the BP algorithm optimized by MEA can obtain better performance than BP with fewer numbers of hidden neurons. It can also be seen that the standard deviation of the generalization performance of BP optimized by MEA is much smaller than for BP, meaning that it may run in a much more stable manner than BP with a faster convergence speed. The relationship between the spent learning time and the number of neurons for BP optimized by MEA and BP is shown in Figure 11.

As shown in Figure 11, the BP algorithm optimized by MEA greatly reduces the training time corresponding to the BP algorithm.

### 4.6. Experiment 6: Test for the Overall Foreign Matter Detection System

We selected 200 bottles of sodium chloride injection to test our detection system, among which 150 samples have typical foreign matter and 50 samples are without foreign matter. To describe the overall system performances, the classification rate for each class and how each of the classes is classified with respect to the others are shown with a confusion matrix in Table 2. Each row *i* of the confusion matrix corresponds to the class of the test sample; each column *j* corresponds to the class decided by the system; and each cell in the table gives the percentage rate of one object of class *i* being classified as class *j*. An ideal system would have a diagonal confusion matrix with 100% for each term of the diagonal.

The misdetection rate and false alarm rate are calculated when unqualified samples are judged as qualified samples and qualified samples are judged as unqualified samples. The misclassification rate is defined as the unqualified samples with foreign matter that are detected accurately, but misclassified.

As shown in the confusion matrix of the detection system, the overall detection system misdetection rate is 1.096% and the false alarm rate is 1.250%. The unqualified samples with three classes of foreign matter are detected at 97.833%, 98.714% and 97.64%, respectively. The qualified sample detection accuracy is 98.75%. The misdetection rate and the false alarm rate are low, and this detection accuracy is satisfactory.

The reasons for misdetection and false alarms are:

1. In a qualified sample, the changes of brightness in bubbles may be falsely detected as unqualified samples with foreign matter.
2. In the process of moving object detection, the objects are easily segmented incompletely; one object broke into several blobs, which confused the BP classifier when judging the class to which it belongs. The segmentation incompleteness may cause the misdetection error and misclassification error, as well as the false alarm.

## 5. Conclusions

This paper mainly describes a new method for the detection and identification of foreign matter in transfusion solution. To avoid large reflective regions on the sidewalls of the plastic bottle, a mixed illumination style is proposed to acquire high contrast images between foreign matter and the background. For the complex background in a transfusion image, which involves the unevenness in the bottle surface, system random noises and the interference of light source instability, Gaussian background modeling is capable of extracting moving objects perfectly compared with the traditional frame difference methods.

Fifteen gray and shape features are extracted for moving objects and are assigned appropriate weights by the ReliefF algorithm according to their contributions to the classification performances. Since the ReliefF algorithm cannot remove redundant features, the BP algorithm test subspace feature dimensions go from one to 15 as the weight values in descending order, and finally, six optimal feature vectors are obtained. The BP algorithm optimized by MEA is able to achieve higher classification accuracy and faster speed for the classification and identification of foreign matter.

The overall system unqualified product detection accuracy is 98.904%, and qualified product detection accuracy is 98.75%, which was tested by many experiments. The misdetection and false alarms are mainly due to the interference of the bubbles. Future work will mainly design more appropriate mechanisms to reduce the production of bubbles and will apply more effective features to distinguish between bubbles and foreign matter.

## Figures and Tables

**Table 1.** The description of the extracted features.

**Number**	**Feature**	**Description**
1	$\overset{-}{\text{f}}=\overset{l-1}{\underset{k=0}{\text{∑}}}kP(k)$	The mean gray value of the object
2	$\sigma^{2}=\overset{l-1}{\underset{k=0}{\text{∑}}}{\left(k-\bar{f}\right)}^{2}P\left(k\right)$	The gray variance of the object
3	$R=1-\frac{1}{1+\sigma^{2}}$	The gray value relative smoothness of the object
4	$u_{3}=\overset{l-1}{\underset{k=0}{\text{∑}}}{\left(k-\overset{-}{f}\right)}^{3}P(k)$	Third moment of the object
5	$U=\overset{l-1}{\underset{k=0}{\text{∑}}}P{\left(k\right)}^{2}$	The uniformity measure of the object
6	$e=-\overset{l-1}{\underset{k=0}{\text{∑}}}P(\text{k})\log_{2}P(k)$	The entropy of the object
7	$A=\underset{\left(x,y\in O\right)}{\text{∑}}1$	The area of the object
8	$\begin{array}{l} R_{\overset{W}{\;}/\underset{L}{\;}}=\frac{W}{L},W=\max(x)-\min(x),\\ L=\max(y)-min(y),x,y\in O \end{array}$	The ratio of length to width of the minimum bounding rectangle of the object
9–15	*_H_*_1_,*_H_*_2_,*_H_*_3_,*_H_*_4_,*_H_*_5_,*_H_*_6_,*_H_*_7_	Seven components of the Hu moments

**Table 2.** Confusion matrix of the detection system.

*i*		**Rubbers**	**Hairs**	**Floating Debris**	**Qualified Products**	**Misclassification Rate**	**Misdetection Rate**
*j*							
Unqualified Products	Rubbers	**97.833**	0.000	1.500	0.667	2.167	0.667
	Hairs	0.141	**98.714**	1.145	0.000	1.286	0.000
	Floating Debris	1.286	0.645	**97.640**	0.429	2.360	0.429
Qualified Products		0.400	0.250	0.600	**98.750**	1.250	0.000
False Alarm Rate		0.400	0.250	0.600	0.000		
